# Directional Amplified Photoluminescence through Large-Area Perovskite-Based Metasurfaces

**DOI:** 10.1021/acsnano.2c09482

**Published:** 2023-01-20

**Authors:** Olha Aftenieva, Julius Brunner, Mohammad Adnan, Swagato Sarkar, Andreas Fery, Yana Vaynzof, Tobias A. F. König

**Affiliations:** † Leibniz-Institut für Polymerforschung e.V., Hohe Straße 6, 01069Dresden, Germany; ‡ Integrated Centre for Applied Physics and Photonic Materials and Centre for Advancing Electronics Dresden (cfaed), Technical University of Dresden, Nöthnitzer Straße 61, 01187Dresden, Germany; § Physical Chemistry of Polymeric Materials, Technische Universität Dresden, Bergstraße 66, 01069Dresden, Germany; ∥ Center for Advancing Electronics Dresden (cfaed), Technische Universität Dresden, 01062Dresden, Germany; # Faculty of Chemistry and Food Chemistry, Technische Universität Dresden, Bergstraße 66, 01069Dresden, Germany

**Keywords:** perovskite nanocrystals, soft lithography, self-assembly, angle-resolved Fourier spectroscopy, Rayleigh anomaly

## Abstract

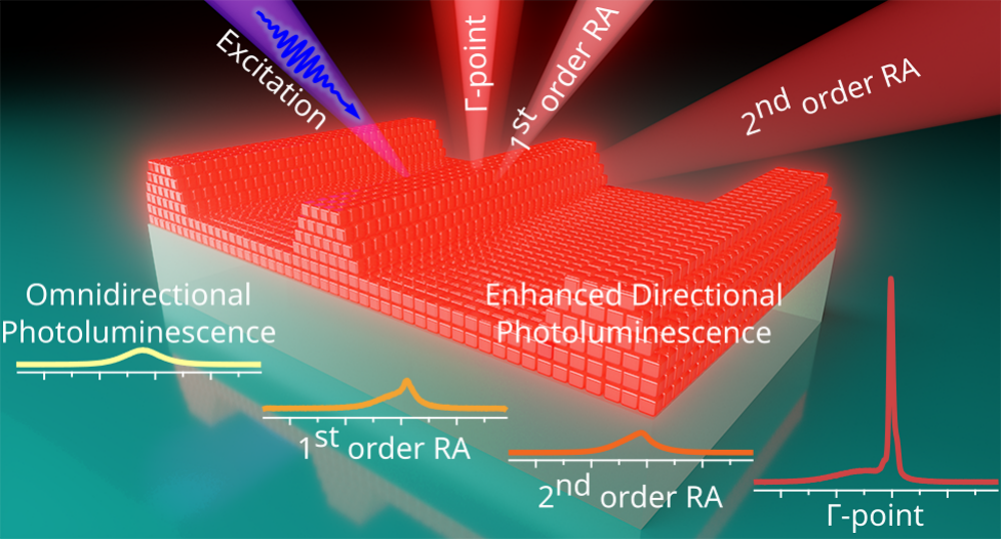

Perovskite nanocrystals are high-performance, solution-processed materials with a high photoluminescence quantum yield. Due to these exceptional properties, perovskites can serve as building blocks for metasurfaces and are of broad interest for photonic applications. Here, we use a simple grating configuration to direct and amplify the perovskite nanocrystals’ original omnidirectional emission. Thus far, controlling these radiation properties was only possible over small areas and at a high expense, including the risks of material degradation. Using a soft lithographic printing process, we can now reliably structure perovskite nanocrystals from the organic solution into light-emitting metasurfaces with high contrast on a large area. We demonstrate the 13-fold amplified directional radiation with an angle-resolved Fourier spectroscopy, which is the highest observed amplification factor for the perovskite-based metasurfaces. Our self-assembly process allows for scalable fabrication of gratings with predefined periodicities and tunable optical properties. We further show the influence of solution concentration on structural geometry. By increasing the perovskite concentration 10-fold, we can produce waveguide structures with a grating coupler in one printing process. We analyze our approach with numerical modeling, considering the physiochemical properties to obtain the desired geometry. This strategy makes the tunable radiative properties of such perovskite-based metasurfaces usable for nonlinear light-emitting devices and directional light sources.

Recently, perovskite nanocrystals gained significant interest from the academic community due to their solution processability, broad band-gap tunability, strong photoluminescence (PL), and high refractive index values.^1−5^ In particular, inorganic perovskites, demonstrating high quantum yields,^6^ are less prone to crystal phase instability^7^ and are less susceptible to degradation under ambient conditions due to the absence of organic components.^8−10^ Cesium lead triiodide (CsPbI_3_) perovskites have been known already for decades but only recently triggered renewed interest due to their potential application in photovoltaics and light-emitting devices.^11−16^ Their synthesis has been explored by numerous groups, enabling the development of a well-established, scalable procedure that yields nanocrystals with strong PL, which is most critical for constructing efficient metasurfaces.^10,13,17,18^ To ensure the long-term phase stability and to prevent the propagation of defects in the crystal lattice, CsPbI_3_ perovskite nanocrystals, capped with oleic acid and oleylamine, can be additionally encapsulated into a polymer matrix that not only protects from humidity^19^ but also creates a uniform refractive index environment that is favorable for the optical performance.^20^ Besides, by controlling the choice of ligands, the nanocrystals can be dispersed in orthogonal solvents, enabling multilayer structures.^21^ Altogether, this makes perovskites ideal building blocks for colloidal light-emitting metasurfaces that represent periodically ordered assemblies on planar surfaces.^22^ The concept of shaping the wavefront of a luminescent material is based on controlling the building blocks of a metasurface at the nanoscale.^23^ The inherent presence of a periodic arrangement on a metasurface implies the emergence of diffractive effects. The classical diffraction theory does not consider the material of the grating itself but rather operates with its geometry and the properties of the surrounding medium.^24^ An extended theory requires, however, the consideration of the grating composition: in the well-known case of metallic corrugated surfaces, diffuse diffraction anomalies originate from the excitation of surface plasmon polaritons.^25^ Similarly, diffraction features couple to the PL when the periodic structure comprises a light-emitting matter and acts by itself as a light source. This notion is supported by the fact that spontaneous emission is not an inherent property of the material but rather arises from the interaction of the material with its local electromagnetic environment.^26^ The resulting radiation pattern of a metasurface is then expected to demonstrate amplification of the emission and directionality-increased photon counts along the directions governed by the diffraction angles.^26−28^

To introduce a periodic structural pattern into otherwise flat, continuous thin films of perovskite nanocrystals, the most straightforward method is through depositing the colloidal nanocrystals onto a prestructured substrate. This approach was previously successfully employed to generate enhanced directional PL,^29,30^ manufacturing nanolasers,^31−40^ photodetectors,^41^ and solar cells.^42,43^ However, such an indirect patterning method lacks the possibility of creating more complex, multicomponent metasurfaces, provides suboptimal electromagnetic energy confinement, and relies on an elaborate substrate preparation.^44^ At the same time, direct patterning through ultraviolet (UV) or electron beam lithography (EBL) increases the risks of material degradation.^45−47^ Some of these approaches have been explored for the patterning of metal halide perovskites, yet they present various challenges, including fabrication on a large scale and material degradation during processing.^48,49^ A more appealing, low-cost, and scalable approach implies direct patterning of perovskite thin films through confinement self-assembly, where the colloidal solution of perovskite nanocrystals (or their precursors that are further turned into a solid crystalline phase) is confined on the substrate by a structured stamp.^22,50−53^ As such, hard silicon (Si) stamps or glasslike molds were successfully employed to create structured metasurfaces with improved crystallinity^54,55^ for modifying the emission properties^38,40,56−59^ and for photovoltaic applications.^60−64^ However, the use of hard stamps requires additional surface modification and operation at high pressure and temperature. As an alternative, soft polymer stamps can be used, since they are low-cost in manufacturing and can be reused multiple times for pattern generation.^44,65^ Until recently, such flexible molds were mostly replicated from compact or digital versatile disks, resulting in templates with only two fixed periodicities of 1.5 μm or ∼750 nm, respectively,^21,64,66−68^ or from masters, produced by cost-inefficient and poorly scalable EBL, reaching the periodicities of few hundred nanometers.^1,28,39,67,69,69−74^ In contrast, by employing laser interference lithography (LIL), one alleviates the aforementioned drawbacks and takes advantage of submicrometer resolution and efficient large-area production.^75^

In this work, we employ a confinement self-assembly technique based on LIL and soft molding to create periodically patterned metasurfaces cost-efficiently over centimeter-scale areas. We provide a universal manufacturing procedure for CsPbI_3_ perovskite nanocrystals capped with a mixture of oleic acid and oleylamine as ligands and dispersed in organic solvents. The resulting metasurfaces feature periodicities ranging from a few hundred nanometers to micrometers. We further provide a profound discussion on the impact of the one-dimensional (1D) periodic structuring on the PL of perovskites, taking into account the angle- and polarization-dependent diffractive behavior of the formed metasurfaces. With the support of angle-resolved spectroscopy, we demonstrate the directionality and multifold amplification of the PL. Note that we intentionally do not use the term amplified spontaneous emission, since the spectroscopic measurements were performed in a constant-wave (cw) mode. We focus our discussion on amplification factors and directionality rather than input–output characteristics. Moreover, we include a grating theory in the analysis that allows for in-depth structural and optical characterization to suggest strategies for the rational design of optoelectronic or photonic devices.

## Results and Discussion

Amplified directional PL is based on the interaction between the emission of the perovskites and the diffraction-related photonic modes. For this, they must overlap energetically, and we show that lattice theory must be closely aligned with fabrication methods to excite the specific radiation patterns. According to the general theory of gratings, a 1D periodic structure exhibits the following diffraction behavior: when an incident light impinges at a certain angle, a discontinuity in the spectrum occurs at a particular wavelength, revealed as an abrupt change in intensity, named thereafter as a Rayleigh anomaly (RA).^24,76,77^ The position of the RAs in the spectrum is governed not only by the geometry of the grating but also by the refractive index of the surroundings, polarization, and the angle of incidence θ of the excitation light
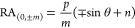
1
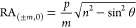
2where *p* is the periodicity of the 1D grating, *m* is the corresponding diffraction order, and *n* is the refractive index of the medium, respectively. Equatuib 1 corresponds to transverse-magnetic (TM) polarization of the incident light, and eq 2 to transverse-electric (TE) polarization.^78^

**Figure 1:**
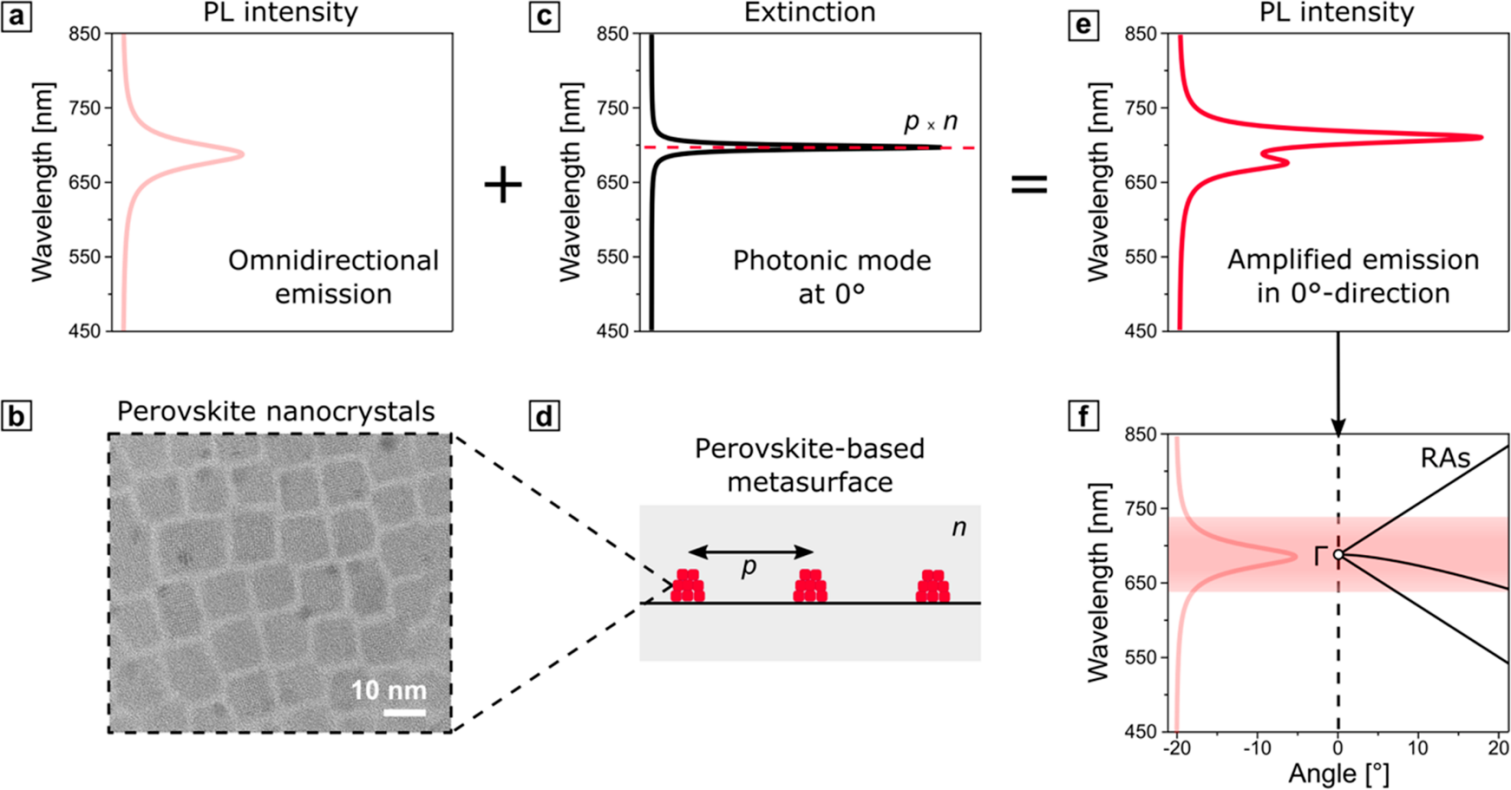
(a) Omnidirectional emission of a thin film of CsPbI_3_ perovskite nanocrystals, shown in a transmission electron microscopy (TEM) micrograph (b). (c) Exemplary extinction spectrum from a grating, schematically shown in (d), under the normal incidence, featuring the photonic mode of the RA, defined by the product of the periodicity *p*, and refractive index of the environment *n*. (e) Modified emission of a perovskite-based metasurface with enhanced PL intensity in the normal direction. The spectra (a, c, e) were calculated analytically via Lorentzian functions and a coupled oscillator model.^81^ For more details, see Table S1 in the Supporting Information. (f) Exemplary dispersion diagram for 1D metasurface with *p* = 450 *nm* and *n* = 1.55. The positions of RAs, marked with solid black lines, and Γ point were calculated analytically with eq 1 and 2. The exemplary emission band of the CsPbI_3_ perovskites, spanning from 640 to 740 nm independently from the angle of incidence, is shown in light red.

The concept through which the PL behavior of perovskite nanocrystals is altered by the presence of 1D gratings and, therefore, the corresponding RAs is schematically depicted in Figure 1. The PL spectrum (shown in Figure 1a), ranging from 640 to 740 nm for the considered CsPbI_3_ perovskites (Figure 1b), can be approximated as a Lorentzian function with a full width at half-maximum (fwhm) from 670 to 708 nm and the peak maximum centered at 689 nm.^79^ An exemplary case of a normal incidence of the excitation light defines the position of narrow-bandwidth RA peaks in the extinction spectrum (Figure 1c) simply via the grating periodicity and the refractive index of the surrounding medium. The overlap of the omnidirectional spectral continuum of the photoluminescent nanocrystals, comprising the metasurface in Figure 1d, and discrete diffraction modes from the periodic structure result in Fano-like interference and promotes amplification of the PL in the direction normal to the metasurface (Figure 1e).^80,81^ The coupling of the photonic modes at off-normal angles can then be accessed through the dispersion diagrams, where the corresponding RAs are spectrally resolved for a specific span of angles (Figure 1f). Nevertheless, the center of the dispersion diagram accommodates the Γ point—the locus of convergence of the photonic RA modes—and therefore is expected to feature the strongest resonant coupling.

**Figure 2:**
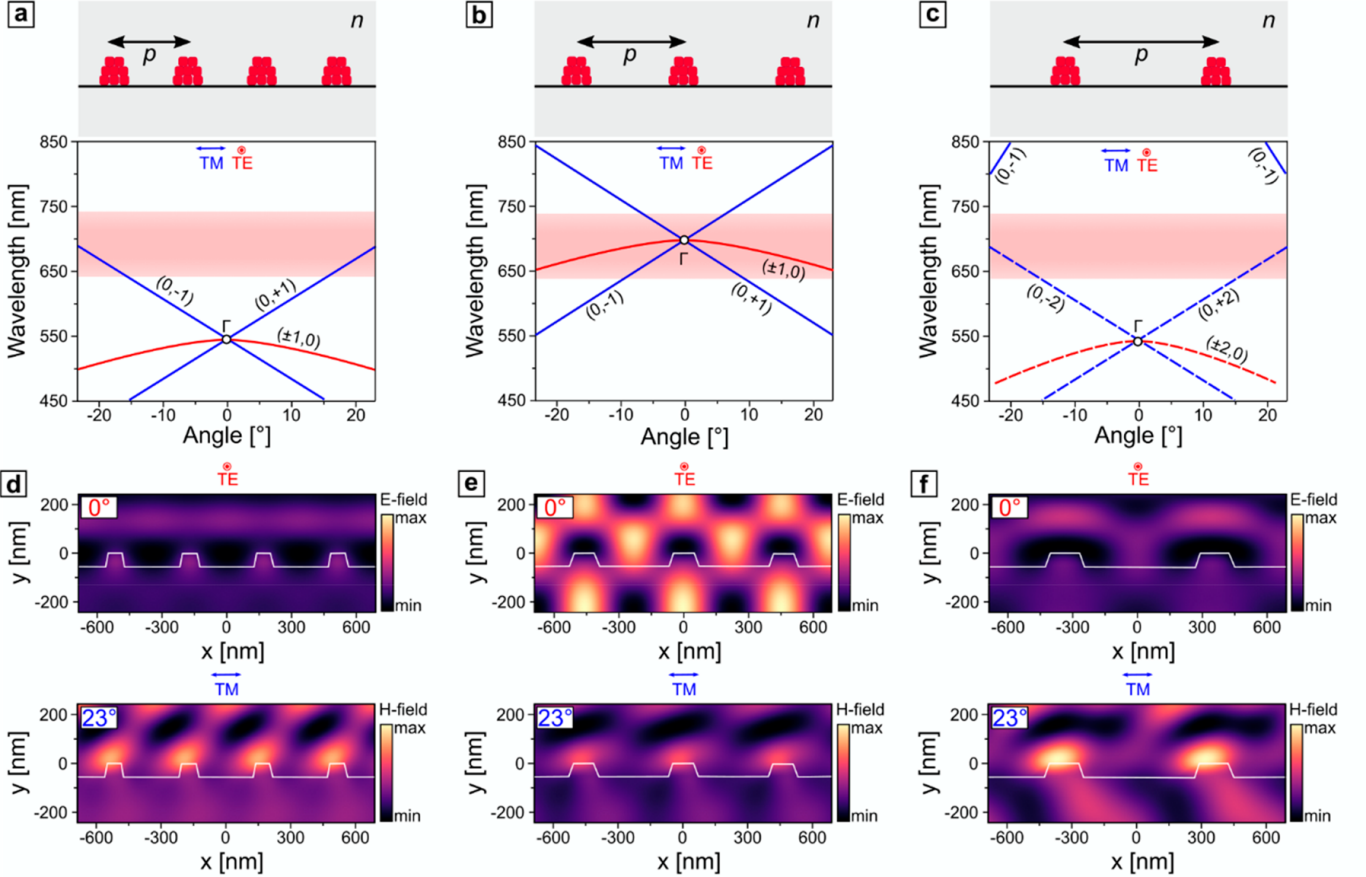
(a–c) Schematic representation of gratings of various periodicities (350, 450, and 700 nm) and corresponding dispersion diagrams. (d–f) Electric *E*-field (top row) and magnetic H-field (bottom row) distribution in three representative periodic structures under normal TE-polarized and angled TM-polarized incidence of a narrow-bandwidth, plane-wave light source centered at the emission maximum (689 nm). Material properties of perovskites for the numerical simulations were determined via spectroscopic ellipsometry, described in detail in Figure S1 in the Supporting Information.

When the parameters are chosen in such a way that the Γ point overlaps with the emission, as shown in Figure 1f, the strongest resonant coupling is expected. On the other hand, the overlap of the RAs at off-normal angles can provide coupling as well and, therefore, enhancement of the PL in particular directions, as shown schematically in Figure 2a–c. In this regard, mode polarization plays a crucial role. TE-polarized modes couple to the PL for a wide angular range, whereas TM-polarized RAs are highly dispersive and overlap with the emission band only at specific angles. Following a particular diffraction order, they demonstrate a stronger angle dependence than TE-modes. The coupling strength in those cases can be qualitatively visualized via the near-field distributions that influence the PL of the light-emitting medium. The spatial confinement of the photonic modes near the Γ point creates a standing-wave pattern (as shown in Figure 2e) and substantial near-field enhancement. Thus, to promote resonant coupling and achieve PL enhancement from the metasurface, the pattern’s periodicity must be chosen in a way that the Γ point matches the PL maximum. On the other hand, to aim at directional PL, one has to match RAs of only TM-polarized modes with the emitted light at the angles of interest (Figure 2a,c). The latter induces the oblique near-field enhancement, as reflected by the magnetic field patterns in the bottom row of Figure 2d,f. As expected, for the cases in which there is no overlap of the emission band and RAs, no field enhancement is observed (bottom row of Figure 2e and top row of Figure 2d,f). Based on these considerations, the parameters of the system can be adjusted to tune the position of the Γ point and either induce the outcoupling of the light in a normal direction or exploit the mismatch between the Γ point and emission band to guide the TM-polarized light at a specific angle.

**Figure 3:**
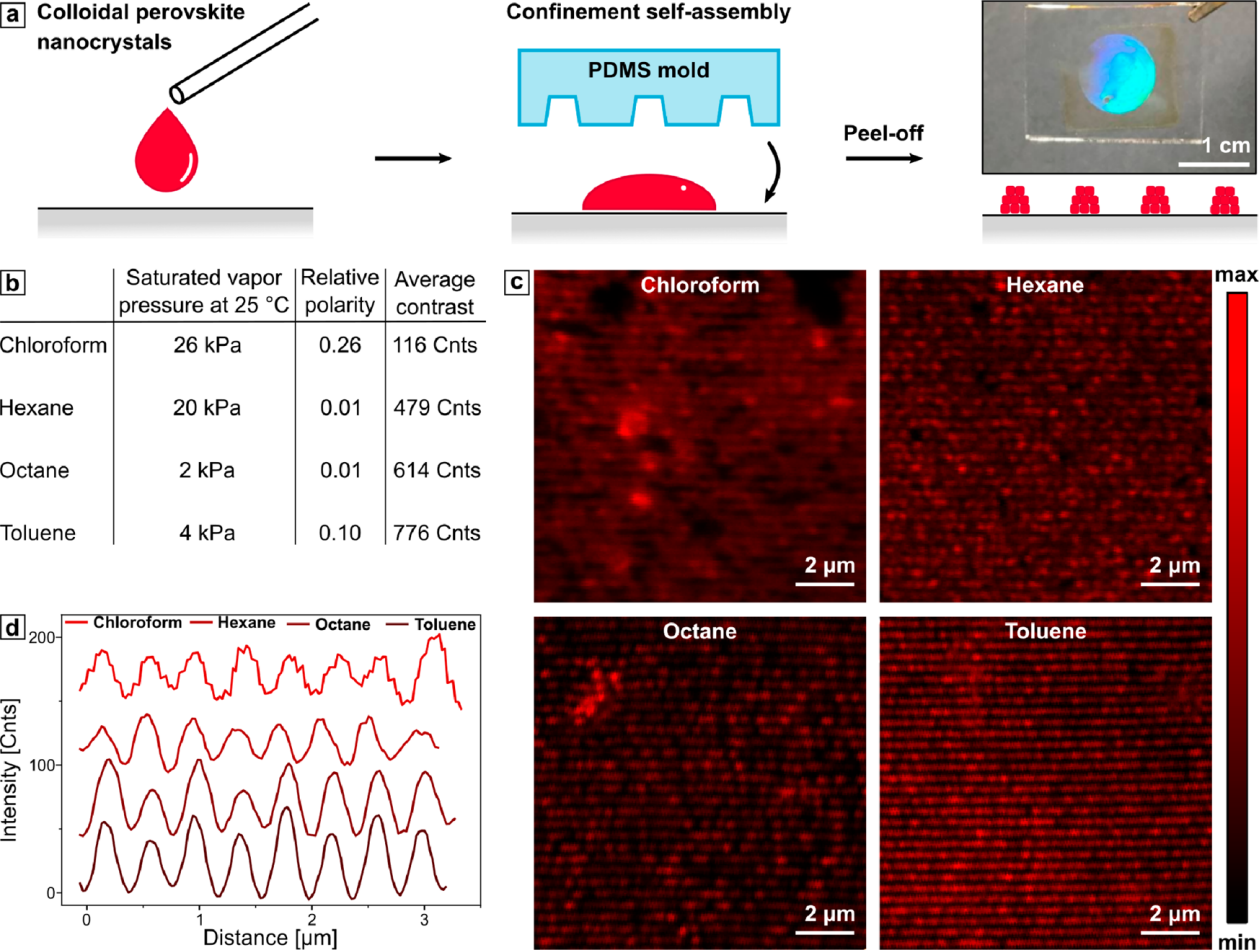
(a) Schematic representation of the assembly process together with a photograph of the large-scale assembly of the colloidal dispersion on the glass substrate under white light illumination. (b) Relevant physical properties of various solvents and the respective average contrast values. (c) CFM images of 1D gratings assembled from 5 mg/mL CsPbI_3_ perovskites in the corresponding solvents. (d) Intensity profiles from the CFM images shifted by 20 counts relative to each other.

To demonstrate light amplification and directionality, we fabricated a set of gratings with specified periodicities predesigned to the emission properties of CsPbI_3_. Confinement self-assembly has been established as a versatile method to arrange nanocrystals into nanostructures over a large area.^82,83^ We provide further details on the fabrication in the Experimental Section. We confined the colloidal solution of perovskite nanocrystals between the substrate and a polydimethylsiloxane (PDMS) mold that acted as a structuring template. One can find further details in the Experimental Section. After evaporation of the solvent through the template, the formed structure represents a reciprocal imprint of the template. Such a technique allows for scalable, single-step production of periodic structures over centimeter-scale areas, as shown in Figure 3a. The organic solvents commonly used for dispersing perovskite nanocrystals are poorly compatible with PDMS. Less polar solvents, such as chloroform, hexane, and toluene, easily penetrate through the porous polymer matrix of PDMS, causing significant swelling and leading to pattern distortions during the assembly.^84^ However, the integrity of the pattern is essential, since its geometry directly affects the diffractive behavior.^85^ To prevent this, one can reinforce the PDMS stamp by changing the ratio of the prepolymer to the curing agent and subsequent thermal treatment.^86^ Another challenge during the assembly is the fast evaporation of the solvent. Thus, one should use solvents with the lowest evaporation rate (lowest saturated vapor pressure). To investigate the solvent effect systematically, we suspended the perovskite nanocrystals in four organic solvents with different evaporation rates: chloroform, hexane, toluene, and octane (Figure 3b).^87^ The assembly quality was then quantitatively accessed through contrast measurements with the help of confocal fluorescence microscopy (CFM) imaging. For contrast image analysis, the average contrast is defined as the average deviation of all points of the intensity profile from a mean line over the evaluation length. The 1D gratings assembled from colloidal solutions are shown in Figure 3c. By comparing the intensity cross-section profiles across the CFM images perpendicular to the grating lines, one expectedly observes the lowest average contrast for the structure assembled using chloroform, where the fast drying of the solvent after the drop-casting results in a patchy pattern (more details can be found in Figure S2 in the Supporting Information). Consequently, assembly with octane with the lowest saturated vapor pressure yielded higher contrast, implicating a more defined grating structure. However, the most distinct contrast and, thus, the best assembly quality was observed in the case of toluene. Although it has a slightly higher evaporation rate than octane, it also features 4 times higher polarity and promotes the colloidal solution spreading over the glass substrate’s intrinsically hydrophilic surface.

Besides the solvent itself, the concentration of the colloidal nanocrystals plays a significant role in the assembly process. Here we used solutions of ∼5 mg/mL that allowed for the assembly of only 1D grating lines, avoiding the accumulation of nanocrystals under the grating structure upon drying. However, by increasing the concentration of the colloidal solution, one can favor the formation of an additional layer below the grating that can be used as a waveguide. In the Supporting Information, we explain this thin film formation as a function of concentration in more detail (in Figure S3). The confinement self-assembly of perovskite nanocrystals, suspended in toluene at low concentrations, yields well-defined 1D gratings.

**Figure 4:**
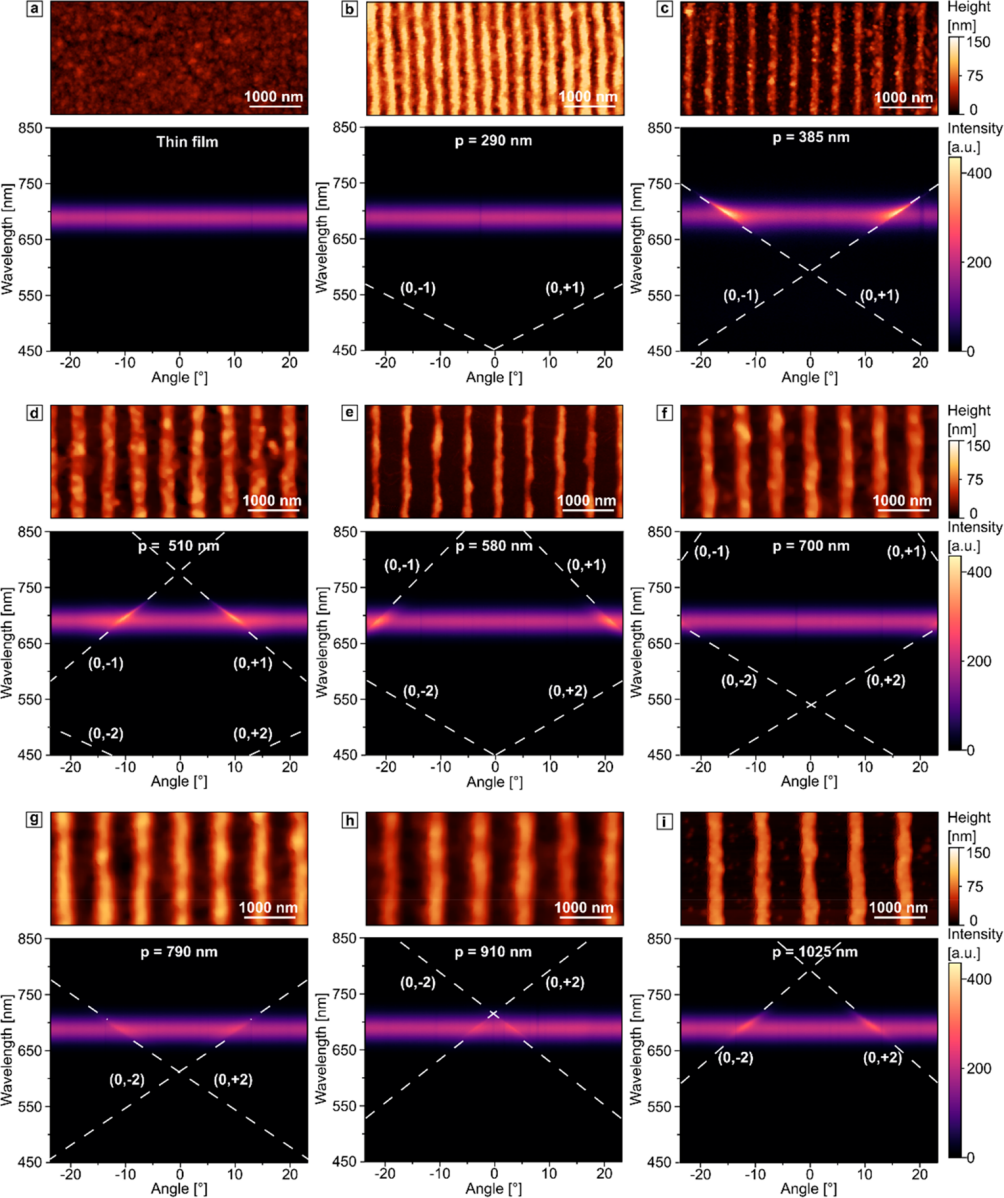
AFM micrographs (top) and dispersion diagrams (bottom) of the (a) thin film of perovskite nanocrystals and 1D gratings with periodicities 290–1020 nm (b–i) under 405 nm laser excitation. The RAs of first and second orders are marked with white dashed lines.

The periodicity can be easily predesigned by choosing PDMS molds with the appropriate geometries, as shown in Figure 4. We also performed an angle-resolved spectroscopy analysis on this set of different periodicities. For all of the gratings, we used solutions at relatively low concentrations (∼5 mg/mL) to minimize the accumulation of nanocrystals under the grating structure upon drying. After assessing the surface profiles of the metasurfaces, the samples were sealed with another glass slide, resulting in a uniform refractive index environment with *n* = 1.55 and ensuring the stability of the prepared samples under ambient conditions (see Figure S4 in the Supporting Information). For this dispersion analysis, we used only TM polarized light. As a reference, we used a flat unstructured thin film. This reference shows an omnidirectional emission, uniform for all detection angles with an intensity maximum at 689 nm. For the grating with a periodicity of 290 nm, the first-order RAs cross the emission band at angles that are out of the observation range. Hence, the angle-resolved PL profile appears similar to that without any pattern. The presence of the structured surface becomes noticeable from 375 nm periodicities onward, where the first-order RAs overlap with emission and induce the PL amplification at particular angles, reflected by a higher number of detected photons along this direction (Figure 4c). Upon increasing the periodicity, the Γ point experiences a red shift, and starting from 685 nm gratings, the second-order RAs begin to interact with the emission spectrum. Since the higher-order RAs feature lower diffraction efficiency,^85^ their interaction with the emission spectrum is marked by less pronounced PL amplification at the corresponding angles. With this self-assembly method from organic solvent, we can change the periodicity gradually and reliably. These results allow us to use the structures for directional photoluminescence of the first- and second-order RAs. Our fabrication method also enables a more accurate step size, which is crucial to observe the interaction of the PL with the high-symmetry Γ point.

**Figure 5:**
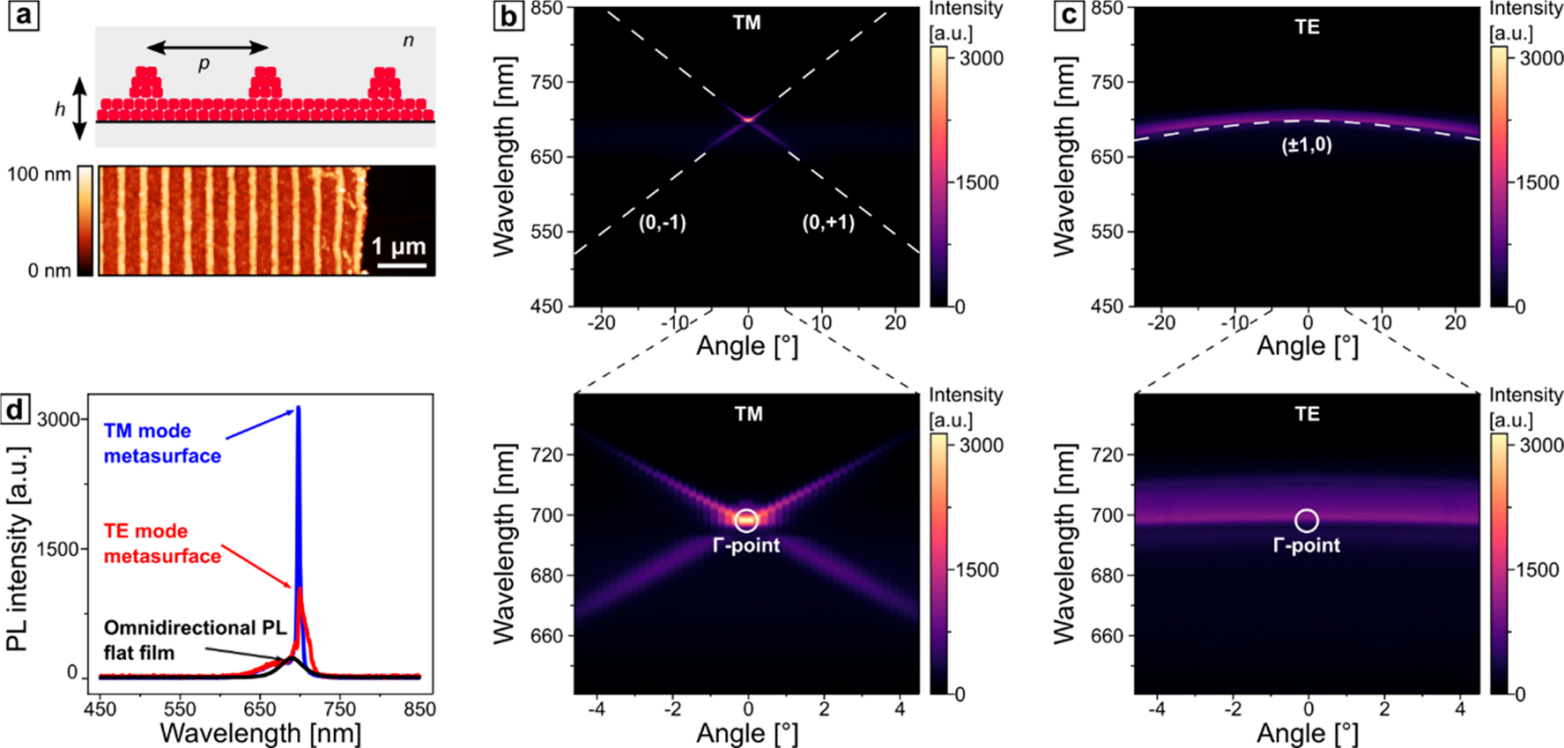
(a) Schematic representation of a printed metasurface, consisting of a thin layer of perovskites and a grating with a periodicity *p* = 450 nm, enclosed in a uniform refractive index environment with *n* = 1.55. (b, c) Dispersion diagrams under TM- and TE-polarized excitation, respectively. The positions of RA for the first orders are marked with white dashed lines. The position of the Γ point is marked in the enlarged insets in the lower panel. (d) Selected PL spectra at 0° for a flat and structured film under TE- and TM-polarized excitations.

Once the center of the Brillouin zone (high-symmetry Γ point) coincides with the emission maximum, strong spatial confinement creates a standing-wave pattern (Figure 2e), perpendicular to the grating, and the emission is amplified. To fabricate such a matching condition, we applied the following procedure: first, we considered the wavelength span of 670–708 nm that corresponds to the fwhm of the emission spectrum from a thin film of perovskites; second, the desired periodicity span was inferred: between 432 and 457 nm (*n* = 1.55). In contrast to the previous section, this particular setting was manufactured from a colloidal solution with a concentration of ∼50 mg/mL, creating a structure with a thin layer under the grating, as shown in the schematic of Figure 5a. Here, we take advantage of the flexibility of our template-assisted self-assembly method and extend the scope to the guided-mode theory. Under the TM and TE polarizations, characteristic first-order RAs were observed (b,c).

As anticipated, the photon counts in the normal direction under both excitation polarizations significantly exceeded the omnidirectional emission from a flat film (Figure 5d). The emission amplification is characterized by decreased spectral width (fwhm) and increased photon counts. The quality factor (*Q*) is defined by the peak position and spectral width quotient. Compared to a reference film, we observe an increase of the *Q* factor by factors of 10 and 2 for the TM and TE modes, respectively (see Table 1 and peak fitting procedure based on nonlinear least-squares minimization in Figure S5 in the Supporting Information). The bandwidth of the PL spectrum can be efficiently narrowed down by 1 order of magnitude, benefiting at the same time from the cost-efficiency and scalability of the experimental approach. The number of emitted photons also increased by a factors of 13 and 4, respectively, being, to the best of our knowledge, the highest amplification factors for the perovskite-based metasurfaces imprinted on a large scale under ambient conditions. The introduced lattice theory states that the TM and TE modes intersect at the Γ point. In the experiment, we find a deviation that leads to a lower amplification for TE polarization. This deviation could be related to different refractive indices for TM and TE polarization, which we discuss in more detail in the following. One can find a comparative amplification analysis in Table S2. As has been extensively discussed elsewhere, the origins of the overall multifold PL amplification are the increase in absorption, strong electromagnetic field confinement that enhances the emission rate, and improved outcoupling from a structured surface.^28,88^

**Table 1:** Comparative Spectral Characterization of the Amplification of the Emission from the Metasurface under TM and TE Polarizations and PL of the Flat Film

	TM mode	TE mode	omnidirectional PL
peak position	698 nm	702 nm	689 nm
fwhm	4 nm	16 nm	38 nm
*Q*	175	44	18
amplification factor	12.9	4.3	

Overall, our soft lithographic self-assembly method allows us to obtain light-emitting metasurfaces with high amplification and *Q* factors without the need to implement photonic or plasmonic crystals or the EBL technique. On the other hand, utilizing an elastic polymer mold allows for multicycle production at a low cost. It also enables producing defined thin layers below the grating that, depending on the thickness, can be exploited either as an additional photon source or as a waveguide under TE polarization. Notably, the change in the thickness of the underlying layer influences the effective refractive index of the surroundings and, therefore, the exact match of the high-symmetry point with the emission band.

**Figure 6:**
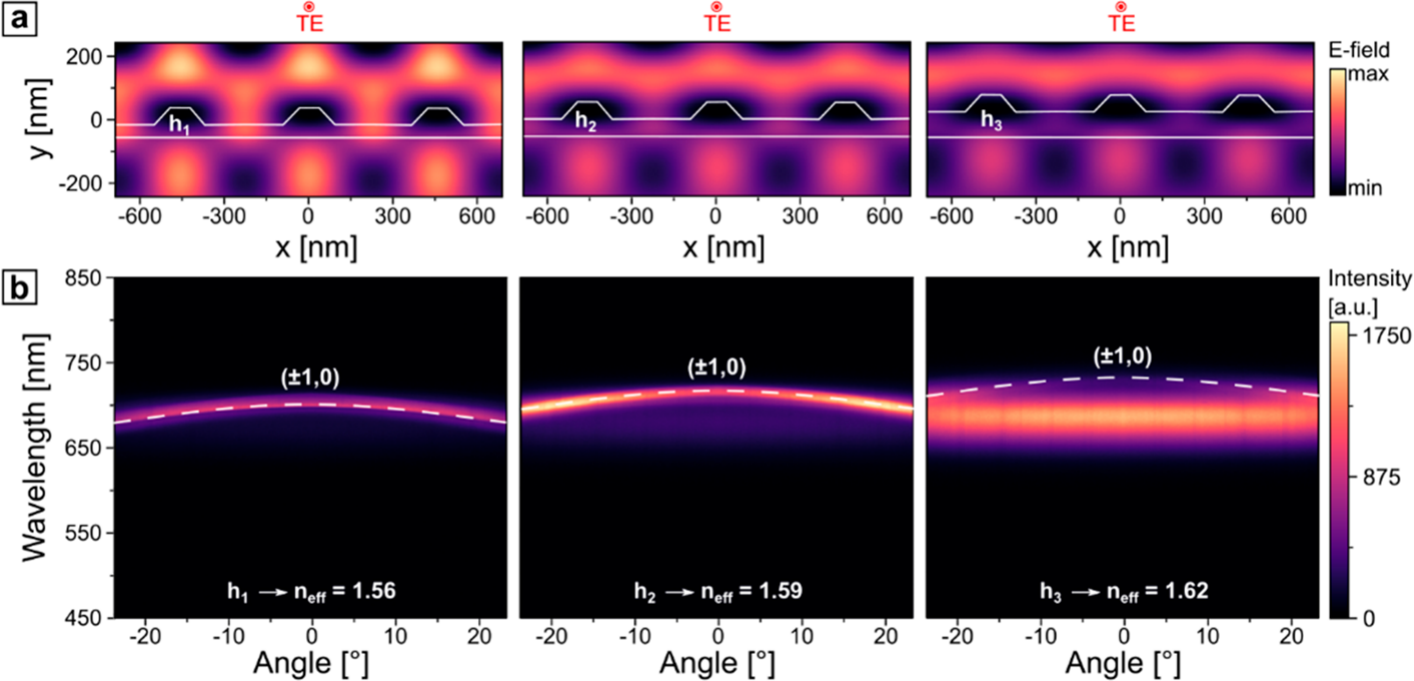
(a) Electric *E*-field distribution in three metasurfaces (*p* = 450 nm) with varied thicknesses of the waveguide-like layer under normal TE-polarized incidence of a plane-wave light source, centered at the RA peak in the emission spectrum. (b) Corresponding dispersion diagrams. The positions of RA for the first order corresponding to the different *n*_eff_ are marked with white dashed lines.

According to the guided-mode theory,^89^ the considered structure can be approximated with a 1D slab waveguide with the effective refractive index ranging from the refractive index of the surrounding medium, *n* = 1.55, and the refractive index of perovskites at 689 nm emission wavelength, *n*_wg_ = 2.04, as can be inferred from the values measured with spectroscopic ellipsometry (see Figure S1). Since the considered structure represents a typical second-order distributed feedback configuration, where the guided mode is scattered out in the direction perpendicular to the plane of the waveguide by the periodic corrugation of the 1D grating residing on top of it, the effective refractive index of the guiding medium *n*_eff_ can be approximated from the Bragg condition
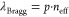
3where λ_Bragg_ is the so-called Bragg wavelength and is determined from the RA peak in the emission spectrum and *p* is the periodicity of the grating. Thus, for the particularly considered case, *n*_eff_ stands at 1.56. Such guided modes are of particular interest for nonlinear optical resonators and are extremely sensitive to the thickness of the waveguide layer. To illustrate this, the dispersion diagrams were recorded at various positions within the sample, where the thickness of the underlying layer was assumed to be different (Figure 6). By fitting the observed TE modes to the RA of the first order, one can derive the *n*_eff_ for each particular case from the Bragg condition, and, consequently, estimate the thickness of the waveguide layer *h* as
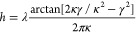
4where λ is the emission wavelength of perovskite nanocrystals (689 nm), , and .^90^

**Figure 7:**
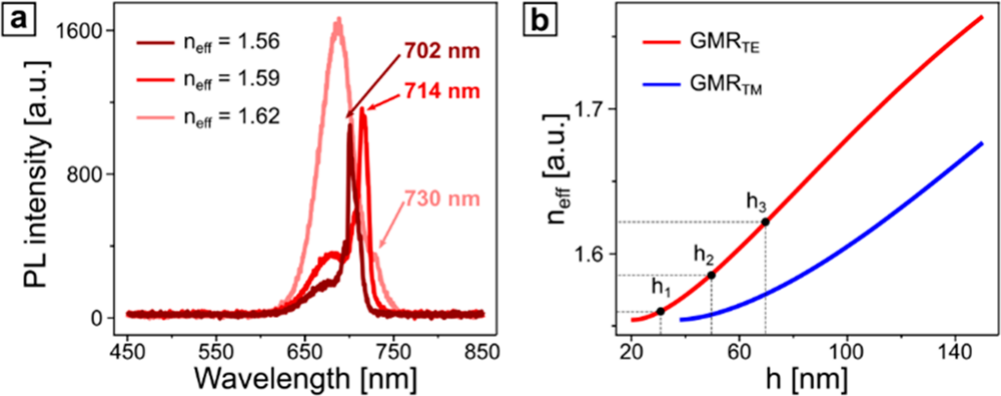
(a) PL intensity spectrum at the Γ point of selected effective refractive indexes (TE mode). (b) Calculated *n*_eff_ for guided TE and TM modes.

The increasing thickness led to higher values of *n*_eff_ that caused a red shift of the TE-polarized guided mode. Substantial electromagnetic near-field enhancement (Figure 6a) promoted energy propagation in the normal direction when it spectrally matched the emission. In contrast, the red-shifted modes overlapped only at higher angles, which correlates with a less pronounced field enhancement (Figure 6b). The overall PL signal increased due to the higher amount of perovskite nanocrystals (see also Figure S6). A systematic study of the cross-section of the angle-resolved diagrams at the Γ point for three different waveguide thicknesses reveals a clear spatial confinement effect for the matching thickness, reflected by the highest *Q* factor of the guided optical mode (see Figure S7), and less pronounced resonances for mismatched cases (Figure 7a). The peak at 689 nm, which corresponds to the emission of perovskites, appears more dominant for the structures with a thicker waveguide-like layer, as expected.

For a continuous range of thickness values, one can calculate the exact *n*_eff_ for the guided mode resonances (GMRs) of both polarizations, as shown in Figure 7b. These results provide an insight into a design procedure, when the geometry of the setup, namely, the thickness of the waveguide layer and periodicity of the grating, is tuned toward the emission maximum. In the thinnest waveguide layer with the thickness *h*_1_, only the TE-polarized guided mode can be excited, contemplating the results, presented in Figure 5, where for the TM polarization the PL of perovskite nanocrystals is altered only by the presence of RAs, whereas for the TE polarization, in addition to RAs, guided modes also interacted with the emission. The validity of such an approach was also confirmed by measuring the actual layer thicknesses with AFM and comparing them to the values estimated by eq 4 and summarized in Table 2 and in Figure S5.

**Table 2:** Calculated *n*_eff_ Values for Guided TE and TM Modes and Comparison of the Calculated Thicknesses *h* of the Waveguide Layer with the Results Obtained from the AFM Measurements at the Corresponding Positions within the Sample

*n*_eff_	*h* (nm)	*h*_AFM_ (nm)
1.56	32.0	30.4
1.59	52.7	54.8
1.62	71.0	71.1

In such a way, by adjusting the *n*_eff_ of the guided modes to the spectral maximum of the emission, one can estimate the appropriate geometry of the waveguide, namely, the thickness of a thin film and periodicity of the grating above, and fabricate the desired structure with the help of soft-lithography-based confinement self-assembly, taking advantage of its high accuracy and robustness. Moreover, such a setup can be efficiently utilized as a thickness sensor to determine the thickness of the underlying layers in a cost-efficient and nondestructive way (see Figure S8).

## Conclusion

In summary, a directional amplification of the photoluminescence from perovskite-based metasurfaces was demonstrated, providing the important prerequisites for a spatially selective outcoupling of emission for light-emitting diodes, backlight displays, and nanometer-sized distributed feedback lasers. The proposed optimization of the confinement self-assembly technique suggests a cost-efficient approach for manufacturing metasurfaces over large areas under ambient conditions, featuring periodicities from hundreds of nanometers up to micrometers, and making it applicable for materials, emitting light in a wide spectral range from blue to the near-infrared regions,^91,92^ or utilizing environmentally friendly quantum dots.^93^ Moreover, we implemented a profound polarization- and angle-resolved spectroscopic examination, supported by the numerical simulations, explaining the origins of the observed optical effects and suggested a strategy for the rational design of the metasurfaces by maximizing the spectral overlap of the emission of perovskite nanocrystals with the diffracted and guided modes.

## Experimental Section

### Synthesis of Perovskite Nanocrystals

The synthesis of the perovskite nanocrystals is done *via* the hot injection method, first published by Protescu et al., with several adjustments.^10^ Before the synthesis, oleic acid (technical grade 90%, Sigma-Aldrich) and oleylamine (technical grade 70%, Sigma-Aldrich) were degassed at 100 °C for 1 h to guarantee high purity of the reactants. Cs-oleate was produced by combining Cs-carbonate with oleic acid. For the preparation of a Cs-oleate solution, 0.407 g of Cs-CO_3_ (TCI, >98%), 20 mL of octadecene (technical grade 90%, Acros Organics), and 1.25 mL of oleic acid were loaded in a two-neck round-bottom flask and degassed for 1 h at 100 °C under vacuum. Thereafter, the flask was filled with nitrogen and heated to 150 °C until all reactants reacted and a clear solution of Cs-oleate was obtained. The Cs-oleate was then stored in nitrogen at 70 °C until use. For the synthesis of CsPbI_3_, 1 g of PbI_2_ (99.99%, TCI) and 60 mL of octadecene were filled in a two-neck round-bottom flask and degassed for 1 h at 120 °C under vacuum. Subsequently, the flask was filled with nitrogen and 6 mL of oleylamine and 6 mL of oleic acid were mixed in a vial and then injected. The flask was again pumped to vacuum for 30 min, until a yellow transparent solution was obtained. Then, the flask was filled with nitrogen and heated to 170 °C. At the target temperature, 4 mL of Cs-oleate was quickly injected into the flask. The solution turned dark red, and after 5 s the reaction was quenched with an ice–water bath. For the purification of the as-prepared CsPbI_3_ nanocrystals, 12.5 mL of the crude solution was mixed with 37.5 mL of methyl acetate (99%, Acros Organics) and centrifuged for 10 min at 6000 rpm. The supernatant was discarded, and the wet CsPbI_3_ pellets were redispersed in 3 mL of hexane (97%, Acros Organics). The solution was again mixed with 5 mL of methyl acetate and centrifuged for 10 min at 6000 rpm. The supernatant was removed, and the precipitates of all tubes were combined into one and dispersed in 25 mL of hexane. This solution was centrifuged for 5 min at 4000 rpm, and this time the supernatant was collected and stored overnight at 4 °C. After that, the solution was centrifuged again for 5 min at 4000 rpm. Finally, the supernatant was collected and dried by using a rotary evaporator. The obtained CsPbI_3_ nanocrystals were dispersed in octane (99%, Acros Organics) at a concentration of 75 mg/mL for further use.

### Laser Interference Lithography

To produce a structured film on the glass substrate, LIL was employed. Right before use, microscopy glass slides were divided into individual pieces (2 × 2 cm) and cleaned with isopropyl alcohol and ultrapure water in a 1:1 ratio by sonication for 20 min at 80 kHz. A positive photoresist (mr-P 1202LIL, micro resist technology GmbH, Germany) diluted with the thinner solution (mat-1050, micro resist technology GmbH, Germany) was spin-coated onto the cleaned substrate and dried under a stream of nitrogen. Optimized spin parameters of 3000 rpm, acceleration of 1000 rpm/s, and total spin time of 33 s produced a thin film of 80 nm thickness, as confirmed by spectroscopic ellipsometry (RC2-DI, J.A. Woollam Co., Inc.) and AFM. The coated substrates were baked at 95 °C for 60 s and further exposed to the 325 nm laser with a dose of ∼12 mJ/cm^2^. The back side of the substrate was covered with black adhesive tape to avoid unnecessary reflections. To develop the exposed photoresist, the sample was submerged into a developer (mr-D 374/S, micro resist technology GmbH, Germany) for ∼1 min, rinsed with ultrapure water, and dried under a stream of nitrogen.

### Confinement Self-Assembly

Produced by LIL, the structured film of a photoresist was replicated using an elastomeric silicone kit (Sylgard 184, Dow Chemicals, USA) with a ratio of prepolymer and catalyst of 5:1 to create a PDMS mold. To further increase the rigidity of the stamp, it was subjected to thermal treatment in an oven at 180 °C for 3 h. Such a process allowed the reduction of swelling of the PDMS mold by chloroform from 125% down to 30%. The resulting mold was trimmed and attached to a weight of 100 g. In the next step, 10 μL of a colloidal solution of CsPbI_3_ perovskite nanocrystals was drop-casted on a cleaned microscopy glass substrate. The weight, together with the PDMS mold attached to it, was immediately placed on the colloidal dispersion to ensure close contact between the mold and the flat surface. The assembly was dried for 1 h at room temperature and a relative humidity of 32%. The stamp was then removed by peeling off. For the CFM measurements and angle-resolved spectroscopy of the metasurfaces with varied periodicities, the stock colloidal solution was diluted to 5 mg/mL with the corresponding solvents. To manufacture the metasurface with a waveguide-like layer, a 50 mg/mL octane-based colloidal solution was used.

### Confocal Fluorescence Microscopy

CFM measurements were acquired with an inverted confocal scanning microscope (MicroTime 200, PicoQuant, Germany) with a 100× air objective (UPLFLN, numerical aperture (NA) 0.9, Olympus, Japan). For excitation, a picosecond pulsed TM-polarized laser diode source (LDH-D-C-405, PicoQuant, Germany) with a center wavelength of 405 nm and a pulse width of 110 ps, driven at a repetition rate of 0.5 MHz, was used. For fluorescence collection, a dichroic mirror (ZT405-442/510rpc-UF3, Chroma, USA), a long-pass filter with a cutoff below 425 nm (FF01–519/LP, Shamrock, USA), and a single photon counting module (SPCM-AQRH, Excelitas, USA) were used. For evaluation, SymphoTime 64 2.3 was used. The fluorescence image scans were recorded at 1 μW excitation power (before the objective) and a dwell time of 2 ms per pixel. The average contrast was calculated as the average deviation of all points of the contrast profile from a mean line over the evaluation length, similarly to the average surface roughness.^94^

### Surface Characterization

The produced line structures were imaged with AFM. The scanning was performed in the tapping mode with silicon nitride probes (typical resonant frequency in the air: 296 kHz). The amplitude set point was adjusted within the range of 100–200 mV at a scanning frequency of 0.5–1 Hz.

### Ellipsometry

To determine the refractive index of the thin crystalline films of perovskites, spectroscopic ellipsometry was performed in the wavelength range from 193 to 1690 nm (combined deuterium/quartz-tungsten halogen lamps) using a spectroscopic ellipsometer (RC2-DI, J.A. Woollam Co., Inc.). The data were acquired in a reflection mode at various angles of incidence ranging from 50 to 70° in 5° steps. To model the refractive index of the substrate, Si with native oxide layer material data was utilized. To determine the refractive index of CsPbI_3_, a general oscillator layer model was implemented within the CompleteEASE (Version 5.19) software. All modeling approximations complied with Kramers–Kronig relations and showed a mean square error (MSE) below 4.

### Angle-Resolved Photoluminescence Spectroscopy

The angle-resolved spectroscopy measurements were performed with a Fourier microscope setup (NT&C, Germany). The sample was illuminated (illumination spot size ∼100 μm) by a continuous-wave polarized laser diode source (LDH-D-C-405, PicoQuant, Germany) with a center wavelength of 405 nm through a bright-field condenser (LWD, NA 0.52, Nikon, Japan). The iris of the light source and aperture of the condenser were fully open to allow illumination at different angles. The back focal plane image (Fourier image) was guided inside the microscope objective (CFI S Plan Fluor ELWD 40×, NA 0.6, Nikon, Japan) into the entrance slit of the spectrometer (IsoPlane 160, Princeton Instruments, USA), opened up to 50 μm. The PL spectrum was directly collected, after filtering the excitation signal with the long-pass filter with a cutoff below 425 nm (FF01-519/LP, Shamrock, USA). The dispersion relation was acquired by collecting the in-plane component of the wave vector , where θ is the angle of incidence/detection, and representing the data in a form of dispersion diagrams with the angular dependences. The intensity of the isotropic part of the collected spectra (taken at the angles away from the RA) was adjusted to the omnidirectional PL from a flat film, manufactured *via* spin-coating (see Figure S1 in the Supporting Information) on a glass substrate.

### Finite-Difference Time-Domain Simulations

A commercial-grade simulator based on the finite-difference time-domain (FDTD) method was used to perform the calculations (FDTD: 3D electromagnetic simulator).^95^ To simulate the optical response, a plane-wave source was used, illuminating the structure at a normal incidence with a polarization angles of 90 and 0° for TE- and TM-polarized light, respectively. The excitation wavelength was selected according to the emission maximum with a pulse length of 25 ps. Perfectly matching layer boundary conditions were used in the *Y* direction, and periodic boundary conditions were used along the *X* axis. The grating lines were represented by trapezoids resembling the experimentally measured grating profiles. To obtain the optical responses of the system, frequency-domain field monitors were used. The dielectric properties of perovskite nanocrystals were imported from the experimentally measured optical constants. For the best simulation stability, the mesh area was set around the existing structure in both principal directions with a mesh step size of 5 nm and the autoshutoff level was set to 10^–6^. The refractive index of the surrounding was set to 1.55. The glass substrate was represented by an “object-defined dielectric” material with a refractive index of 1.5, extending through the bottom boundary of the simulation unit cell in the *Y* direction.

## Supporting Information Available

Detailed working principle of a light-emitting metasurface, optical properties of thin films of perovskite nanocrystals, effect of the solvent on the quality of the assembly, effect of the concentration of the colloidal solution on the assembly, stability of the thin perovskite films under ambient conditions, peak fitting procedure, thickness variation of the waveguide-like layer, thickness sensor setup, comparative study on PL amplification, and thickness variation of the waveguide-like layer (PDF)
