# Supplementary material for: Directional Amplified Photoluminescence through Large-Area Perovskite-Based Metasurfaces
Source: ACS Nano. 2023 Jan 20;17(3):2399–410. doi: 10.1021/acsnano.2c09482 (PMC9955732; doi:10.1021/acsnano.2c09482)
Supplement: Supplementary file 1 — nn2c09482_si_001.pdf [file nn2c09482_si_001.pdf]

SUPPORTING INFORMATION

Directional Amplified Photoluminescence through Large-Area Perovskite-Based Metasurfaces

*Olha Aftenieva,[a] Julius Brunner,[b] Mohammad Adnan,[a,c] Swagato Sarkar,[a] Andreas Fery,[a,d] Yana Vaynzof,[b,e] and Tobias A.F. König[a,e,f]**

[a] Leibniz-Institut für Polymerforschung e.V., Hohe Straße 6, 01069 Dresden, Germany

[b] Integrated Centre for Applied Physics and Photonic Materials and Centre for Advancing Electronics Dresden (cfaed), Technical University of Dresden, Nöthnitzer Straße 61, 01187 Dresden, Germany

[c] New address: Physikalisches Institut, WWU Münster, Wilhelm-Klemm-Straße 10, 48149 Münster, Germany

[d] Physical Chemistry of Polymeric Materials, Technische Universität Dresden, Bergstraße 66, 01069 Dresden, Germany

[e] Center for Advancing Electronics Dresden (cfaed), Technische Universität Dresden, 01062 Dresden, Germany

[f] Faculty of Chemistry and Food Chemistry, Technische Universität Dresden, Bergstraße 66, 01069 Dresden, Germany

*Corresponding e-mail: yana.vaynzof@tu-dresden.de, könig@ipfdd.de

Detailed working principle of a light-emitting metasurface

The analytical representation of two coupled oscillators, described by Lorentzian functions, can be given as follows:

$$\sigma_{ph} = \omega^4 \left| \frac{1}{\omega_{ph}^2 - \omega^2 - i\omega\gamma_{ph}} \right|^2 \#(1)$$

$$\sigma_{per} = \omega^4 \left| \frac{1}{\omega_{per}^2 - \omega^2 - i\omega\gamma_{per}} \right|^2 \#(2)$$

$$\sigma_{meta} = \omega^4 \left| \frac{\omega_{per}^2 - \omega^2 - i\omega\gamma_{per}}{(\omega_{per}^2 - \omega^2 - i\omega\gamma_{per})(\omega_{ph}^2 - \omega^2 - i\omega\gamma_{ph}) - g^2\omega_{per}\omega_{ph}} \right|^2 \#(3)$$

where $\sigma_{per/meta}$ is the emission cross-section of the unstructured perovskite thin film and the perovskite-based metasurface, respectively; σ_{ph} - extinction cross-section of a photonic mode, introduced via the structuring of a thin film; ω is the photon frequency; γ denotes damping and g refers to the coupling strength of the two oscillators.[1]

Table S1: Parameters for the coupled oscillator model.

	ω [eV]	γ [eV]	g [eV]
Perovskites	1.800	0.099	6
Photonic mode	1.776	0.016	

The resonant frequencies $\omega_{per/ph}$ match the experimentally measured emission maximum wavelength (689 nm) of the unstructured perovskite thin film and the photonic mode of a metasurface with the periodicity of 450 nm in a uniform refractive index environment of 1.52 (698 nm). The damping factors are represented by a full width at half maximum (FWHM) of a corresponding Lorentzian function: for perovskites – 38 nm and 6 nm – for a photonic mode, inferred from fitting the experimental data. The coupling factor was chosen empirically to match the actual line shape of the emission spectrum of the metasurface. Presented analytical model

serves the visual purpose only and cannot be used for a quantitative characterization of the actual coupling of the resonant modes within a metasurface. Calculated cross-sections are depicted in Figure 1 in the main text.

Optical properties of bulk perovskite material

To determine the refractive index of $CsPbI_3$, spectroscopic ellipsometry measurements were performed. For this, a thin film of perovskites was prepared on the silicon (Si) substrate by the spin-coating technique. A ~50 mg/mL solution was drop-casted in a dynamic coating mode at 3000 rpm. The thickness and roughness of the thin film were determined with the help of AFM measurements (41 ± 7 nm) and were then utilized as the starting parameters for the fitting procedure of the ellipsometric data. The initial fitting range was set to a transparent region of 700 – 1300 nm, where the experimental data was approximated with the B-spline model. After expanding to a full wavelength range, the B-spline model was parameterized by a set of oscillators, matching the characteristic peaks in the absorption spectrum.[2]

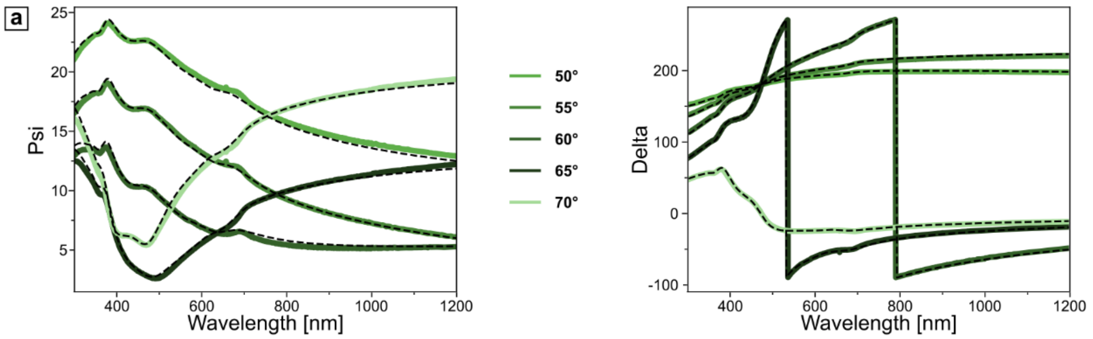

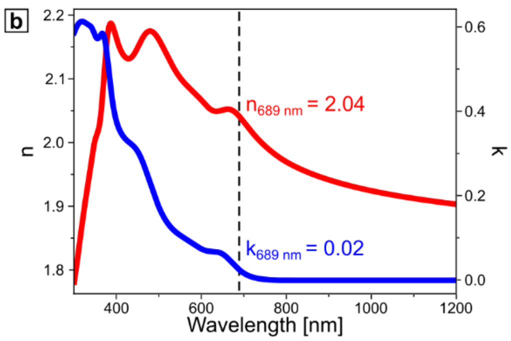

c

Oscillator parameters

	Amp_n	Br_n [eV]	E_{0n} or E_n [eV]	E_{gn} [eV]
1: T-L	19.02 eV	3.00	3.65	1.57
2: Gau	0.25	0.66	3.80	-
3: Gau	0.11	0.22	3.59	-
4: Gau	0.51	0.23	3.33	-
5: Gau	0.28	0.37	2.71	-
7: Gau	0.12	0.18	1.91	-
6: Gau	0.09	0.36	2.12	-

Figure S1. Ellipsometry characterization of the thin film of $CsPbI_3$ nanocrystals: (a) psi and delta ellipsometric parameters for angles in the range of 50° - 70° together with an appropriate model fit with the MSE<3.5. (b) Wavelength-resolved optical (real n and complex k) constants. (c) Oscillator parameters of the model fitted to the psi and delta parameters.

Effect of the solvent on the quality of the assembly

To assess the quality of the assembled metasurfaces, we performed spectroscopy and time-correlated single-photon-counting (TCSPC) in addition to the contrast measurements, as shown in Figure S2. For spectroscopy (Figure S2a), the sample was excited under a constant illumination at 405 nm and the signal was recorded by the spectrometer Newton 920 (Oxford Instruments, UK). With the help of TSCPC, we calculate the photoluminescence quantum yield (PLQY) *via* lifetime measurements. We successfully applied this approach in earlier work to underline the difference in the quantum efficiency in a liquid colloidal solution and thin films.[3] Our semiquantitative approach has proven more reliable than determining the PLQY of nanostructures using the integration sphere. Briefly, we assumed the radiative decay rate to be constant in both solid and liquid samples, which results in a direct dependency of PLQY on the lifetime. For comparison, the fluorescent decay was measured in liquid (octane) and metasurfaces, assembled from chloroform-, hexane-, octane-, and toluene-based colloidal solutions. For measuring lifetime in liquid, a highly diluted colloidal solution was kept in chambered coverglass wells (NuncTM Lab-Tek). The measurement was performed with a single-photon counting module (SPCM-AQRH, Excelitas, USA) and calculated via SymphoTime 64 2.3 software by fitting the intensity decay with the 3-exponential deconvolution and using the computed instrument response function (IRF). The absolute PLQY in liquid (56%) was measured with with a homebuild setup, where the sample was placed in a integrating sphere

and excited by a 405 nm laser at 10 mW. The PL was then recorded by a spectrometer (Ocean Optics QE65 Pro). The absolute PLQY was used as a reference to estimate the PLQY in assembled metasurface.

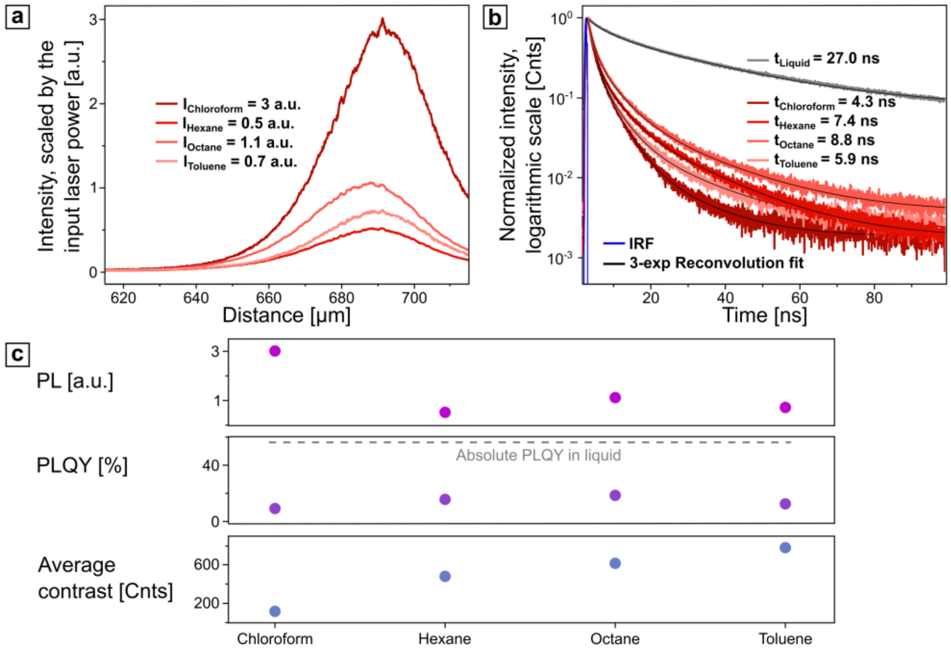

Figure S2: (a) PL and (b) average lifetime measurements of four metasurface, assembled from different organic solvents. The instrument response function (IRF), used for fitting the decay data is shown in blue. (c) Summarized PL, PLQY and contrast characteristics of the produced metasurfaces. The average values (Mean) and their standard errors (SE) are shown in the corresponding colors.

The strongest PL signal showed the metasurface assembled from the chloroform-based colloidal solution (Figure S2a). The latter can be attributed to agglomerates with more light-emitting nanocrystals in the observation range than well-formed grating. We explain the apparent decrease in a lifetime in solid samples (Figure S2b) and, therefore, in PLQY, by the dense spatial confinement of nanocrystals upon drying that induces resonant transfer effects.[4] This

corroborates the fact that the chloroform-based metasurface, with the lowest contrast and high level of aggregation, showed the lowest PLQY value. Moreover, the lifetime measurements were performed after the samples were stored unprotected in ambient conditions for several days, which strongly affected the PL efficiency. Nevertheless, such reduced PLQY values were sufficient to induce PL and measure the contrast ratio from the CFM images. This study provides extensive information on how well-defined the grating lines are. On the other hand, PL and PLQY measurements indicate more on the amount and the integrity of the light-emitting material.

Effect of the concentration of the colloidal solution on the assembly

The concentration of the colloidal nanocrystals plays a crucial role in the assembly process. By adjusting the concentration, one can achieve different thicknesses of the flat layer assembled upon drying under the periodic pattern (Figure S3a). The unavoidable initial drying of the solvent during the time gap between the drop-casting of the colloidal solution and stamp placement results in a thin film formation. The thickness of this pre-assembled layer is governed by the concentration and the spreading of the colloidal solution, and, thus, varies on the millimeter-scale over the structured area. Nevertheless, such variations appear within certain concentration ranges and can be visually estimated through the color appearance of the produced structured pattern; the darker color indicates the higher layer thickness, as demonstrated in Figure S3b-d.

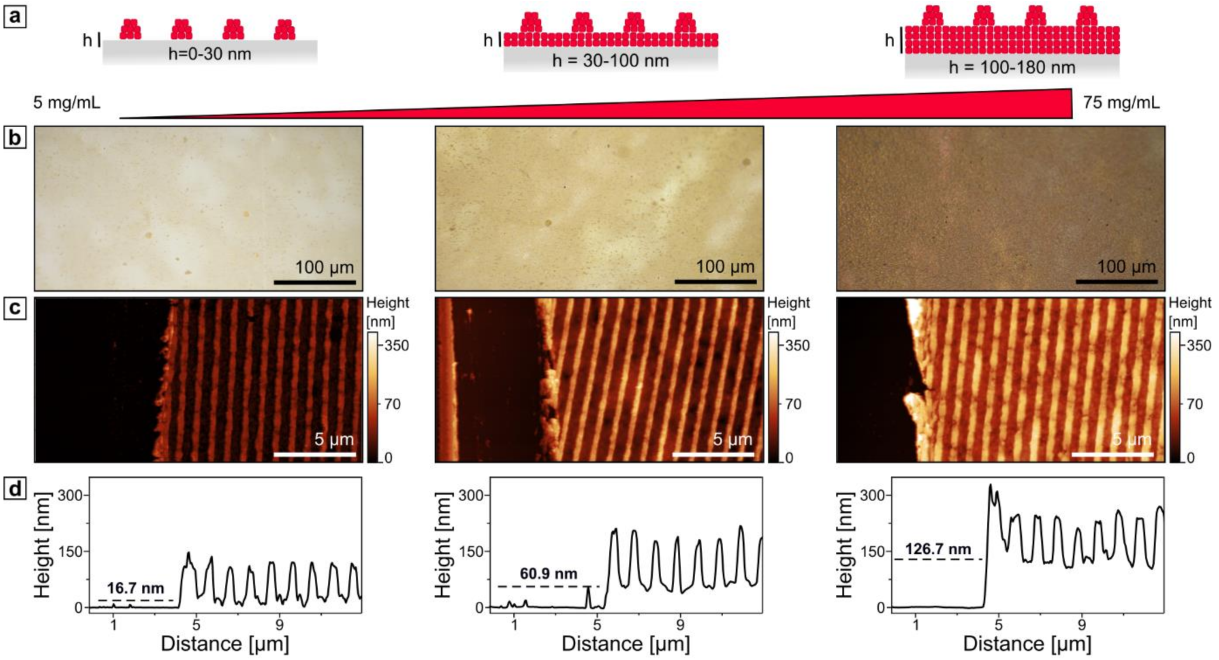

Figure S3. (a) Schematic representation of structured films with different thicknesses of the underlying layer depending on the concentration of the colloidal solution. (b) Bright-field microscopy images of three representative structured surfaces with the concentration increasing from left to right. (c) AFM micrographs and (d) corresponding height profiles were measured across the pattern perpendicular to the grating lines.

Stability of the thin perovskite films in the ambient conditions

To study the stability of the perovskite nanocrystals on a solid substrate, we first prepared the unstructured films *via* dynamic coating of the colloidal solution at ~80 mg/ml in octane on a glass substrate at 3000 rpm. The thin films were then left in ambient conditions at a relative humidity of 32% without light protection. We considered three configurations of thin films, which are as follows (see also **Figure S*4***): (a) completely unprotected, (b) covered by fluoropolymer CYTOP (AGC, CTL-809M), and (c) sealed with another glass slide *via* the adhesive polymer NOA 61 (Norland Products, USA).

For the configuration (b) we used the following fabrication parameters: 1 mL of CYTOP was dissolved in 333 uL of CT-Solv.180 (AGC) and 70 µl were deposited by dynamic coating at 500 rpm for 3 s and 1500 rpm for 60 s. After that, the sample was annealed at 90 °C for 5 min.

On the other hand, the encapsulation (c) was done by drop-casting NOA 61 and immediately placing the sealing glass slide. The sample was then subjected to UV irradiation at 254 nm with a power of 12 W (M&S Laborgeräte) for 10 min. Finally, we assessed the stability of the perovskite nanocrystals by recording the absorption spectra over time.

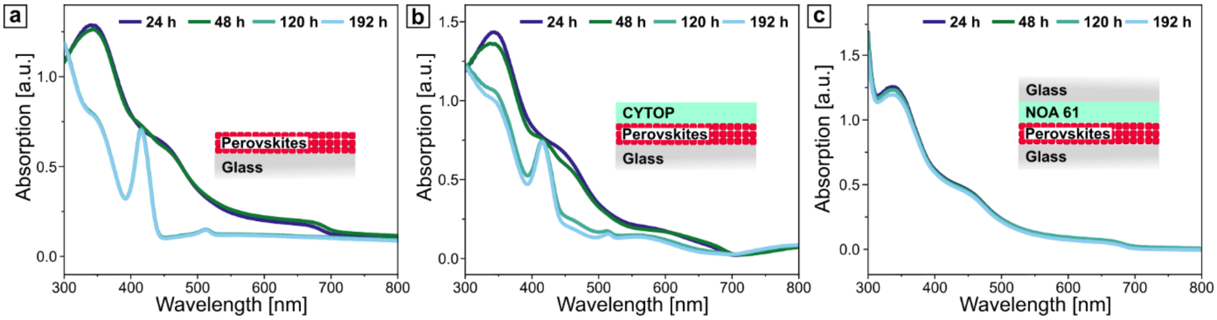

Figure S4: Absorption spectra of thin, unstructured perovskite films (a) without any protective upper layer, (b) covered with CYTOP, and (c) encapsulated with NOA 61 and additional glass slide. The measurements were taken 24, 48, 120, and 192 h after preparation.

Typical onset at ~700 nm, corresponding to the bandgap of $CsPbI_3$, was present in all configurations after the fabrication. The thin film, covered with CYTOP, featured additional scattering at higher wavelengths due to the higher roughness of the film. On the contrary, we do not observe parasitic scattering in the thin film encapsulated in a uniform refractive index environment with NOA 61. The unprotected sample and the sample with CYTOP as a protective layer showed distinctive signs of degradation after 120 h, whereas the encapsulated thin film remained intact after 192 h. The latter indicates that the material still had semiconducting

properties to absorb photons after eight days. From these results, we can conclude that the PL properties remain unchanged for an extended duration.

Peak fitting procedure

The fitting procedure was performed with the help of the python-based script using the *lmfit* package based on non-linear least-squares minimization.[5] The PL spectra were approximated with the help of Lorentzian functions, matching the main PL peaks.

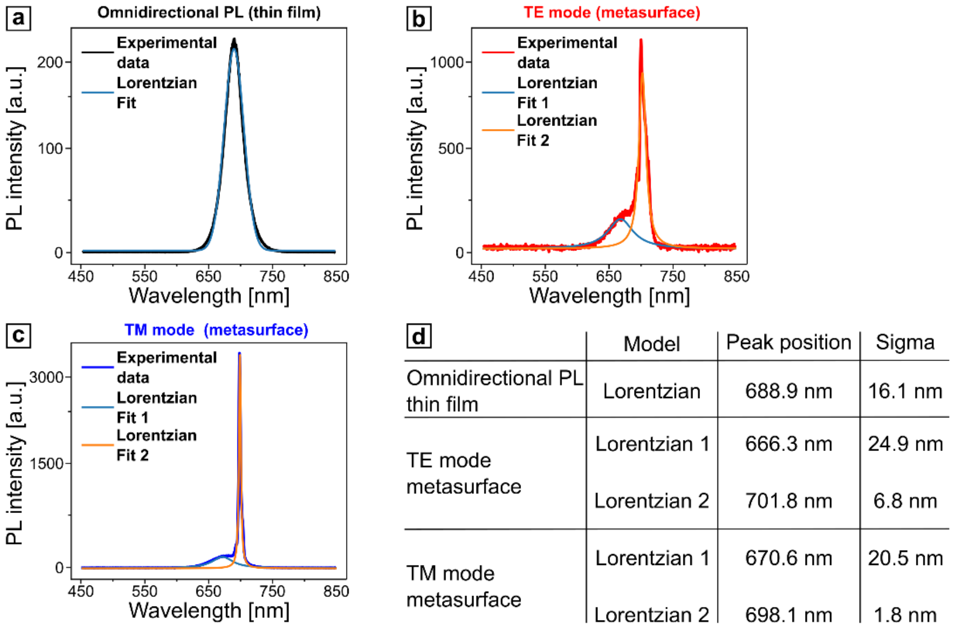

	Model	Peak position	Sigma
Omnidirectional PL thin film	Lorentzian	688.9 nm	16.1 nm
TE mode metasurface	Lorentzian 1	666.3 nm	24.9 nm
	Lorentzian 2	701.8 nm	6.8 nm
TM mode metasurface	Lorentzian 1	670.6 nm	20.5 nm
	Lorentzian 2	698.1 nm	1.8 nm

Figure S5. Detailed spectral analysis of the PL at 0° detection angle (Γ-point) for (a) uniform PL from a flat film; and a periodic 1D structured metasurface under (b) TE and (c) TM polarizations. (d) Summary of the fitted peak parameters.

Comparative study on PL amplification

Table S2. Comparison of the amplification factors in structured perovskite metasurfaces, depending on the manufacturing technique.

Amplification factor	Processes involved	Perovskites	Pattern geometry	Minimum size/periodicity	Reference
70	Hard NIL & annealing	$MAPbI_3$-based composite	Lines and holes	300 nm	[6]
24	Reactive ion etching	$CsPbBr_{2.75}I_{0.25}$	Holes	280 nm	[7]
21	Infiltration to a photonic crystal	$MAPbBr_3$	3D opal	200 nm	[8]
13	Soft NIL	$CsPbI_3$	Lines	300 nm	This work
12	Combination with a plasmonic metasurface	$MAPbI_3$	Holes	200 nm	[9]
8	Hard NIL	$MAPbI_3$	Lines	350 nm	[10]
7	Template-confined epitaxial growth	$MAPbBr_3$	Lines	600 nm	[11]
5	Combination with a plasmonic metasurface	$MAPbBr_3$	Randomly distributed disks	-	[12]
5	Soft NIL & annealing	$CsPbBr_3$	Cylinders & Cones	400 nm	[13]
4	Combination with a plasmonic metasurface & reactive ion etching	$MAPbBr_3$	Randomly distributed disks	-	[14]
4	Thermal hard NIL	$MAPbI_3$	Triangles	400 nm	[15]
4	Infiltration to a photonic crystal	$CsPbBr_3$	Kagome lattice	250 nm	[16]

3	Focused ion beam milling	$MAPbI_3$	Lines & holes	300 nm	[17]
3	Thermal hard NIL	$MAPbI_3$	Lines	600 nm	[18]
2	Soft NIL & annealing	$MAPbI_3$	Lines and holes	1000 nm	[19]
<2	Reactive ion etching	$CsPbBr_3$	Holes	157 nm	[20]
<2	Hard NIL & annealing	$MAPbI_3$	Lines	600 nm	[21]
<2	Focused ion beam milling	$MAPbBr_3$	Cylinders	400 nm	[22]

Thickness variation of the waveguide-like layer

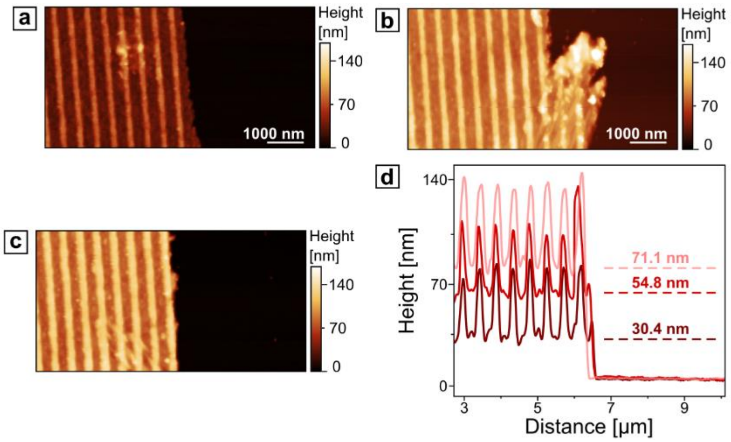

Figure S6. (a-c) AFM micrographs of three different measurement positions on the 450 nm periodic structured film, where the corresponding angle-resolved spectroscopic measurements were taken. (d) Thickness cross-section profiles were taken across the AFM micrographs, perpendicular to the grating lines.

The spectral shift of the resonant upon increasing thickness was analyzed via the fitting procedure with the help of the python-based script using the *lmfit* package based on non-linear

least-squares minimization.[5] The PL spectra were approximated with the help of Lorentzian functions, matching the prominent PL peaks. The guided mode corresponds to the second Lorentzian function at longer wavelengths.

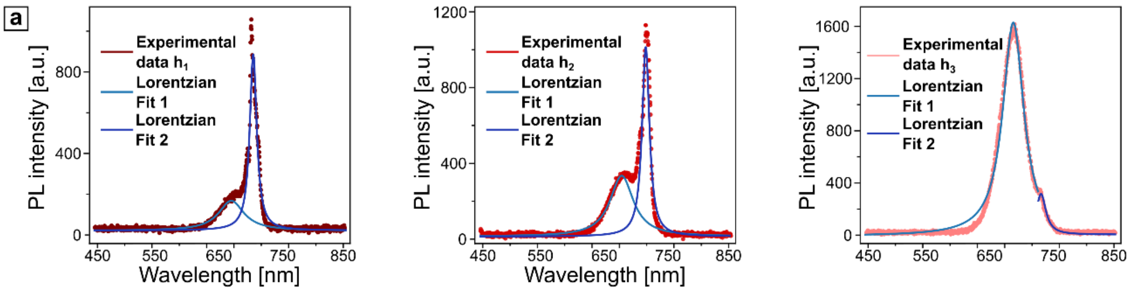

b

	Model	Peak position	Sigma
h_1	Lorentzian 1	666.3 nm	24.9 nm
	Lorentzian 2	701.8 nm	6.8 nm
h_2	Lorentzian 1	675.2 nm	23.1 nm
	Lorentzian 2	714.4 nm	7.2 nm
h_3	Lorentzian 1	685.5 nm	20.7 nm
	Lorentzian 2	730.4 nm	9.6 nm

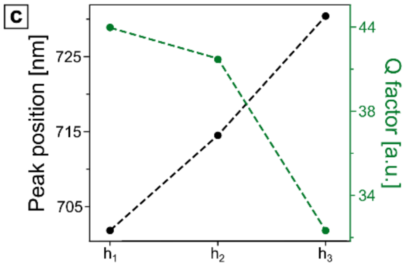

Figure S7: (a) Detailed spectral analysis of the PL at 0° detection angle (Γ-point) under TE-polarized light from metasurfaces with varied thicknesses of the waveguide-like layer. For the metasurface with waveguide thickness h_3, the fitting procedure was performed separately for two spectral regions: 445 – 725 nm and 725 – 852 nm, to better resolve the resonant peaks. (b) Summary of the fitted peak parameters. (c) Spectral position and Q factor of the guided modes.

Thickness sensor setup

To illustrate the practical usability of such perovskite-based metasurfaces, we suggest a setup that can be employed as a thickness sensor for thin films on glass substrates. Polyethyleneimine (PEI) was chosen as an exemplary material, widely used for a defined spacer or adhesion promoter on glass surfaces.[23] The proposed setup includes a perovskite-based metasurface assembled on an elastic PDMS substrate that can be placed on a sample of interest. Using a flexible substrate ensures close contact with the analyte material and allows for multiple usage

cycles.[3] As a proof-of-concept, we provide the FDTD-based numerical simulations to demonstrate the exceptionally high sensitivity of such a setup compared to a thin-film configuration without a metasurface, as depicted in Figure S8a,b. The frequency domain field monitor, located in the glass substrate, allows recording the transmission as a function of wavelength and observing the spectral shift of the optical mode with a changing thickness of the layer of interest. The narrowband optical modes that arise due to the presence of a metasurface also demonstrate superior sensitivity S estimated as the slope when plotting the resonance peak as a function of the thickness values (Figure S8c). The calculated figure of merit (FoM) values are two orders of magnitude higher when perovskite-based metasurface is included, which outlines the advantage of the suggested setup for sensing purposes. Experimentally, one can place the metasurface on a flexible substrate on a solid transparent substrate containing a thin film of interest with an unknown layer thickness. By illuminating such a configuration with a broadband light source and recording the position of the resonant optical mode, one can estimate the thickness given the known optical parameters of the materials. Alternatively, the structure can be excited by a narrowband light source, inducing PL of perovskites, and allowing for tracking the position of the resonant mode in the emission spectrum.

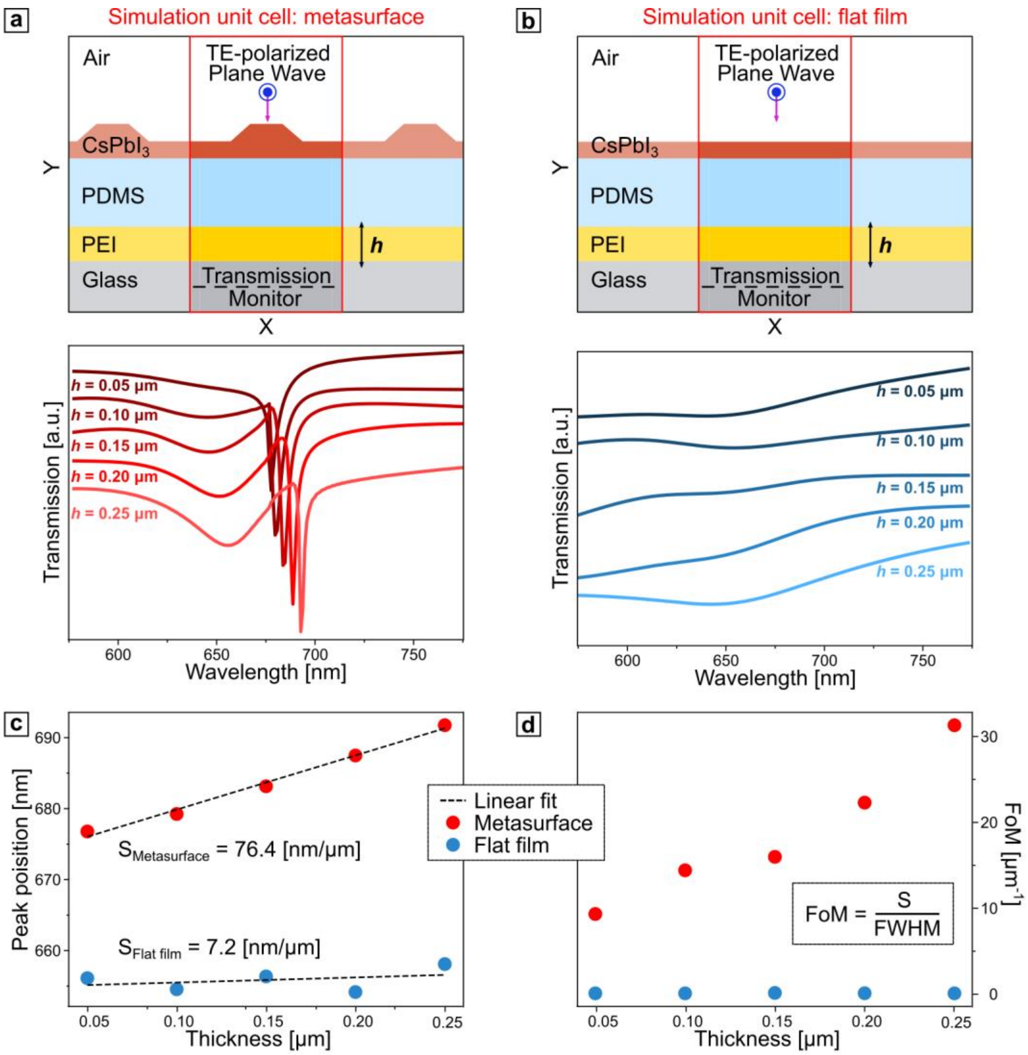

Figure S8: Top row: simulation setup of the metasurface-based sensor (a) and flat film (b). Bottom row: simulated transmission spectra for the corresponding sensor setups. The spectra were shifted relative to each other for better visibility. (c) Sensitivity of the resonant mode for the metasurface- and the flat film-based sensor. The peak wavelength shifts linearly with the increasing thickness of the analyzed PEI layer. The slope, representing the S value, was extracted from the linear fitting of the simulated data with the python-based script using the polyfit function. (d) Calculated figure of merit (FoM) values.

REFERENCES

(1) Wu, X.; Gray, S. K.; Pelton, M. Quantum-Dot-Induced Transparency in a Nanoscale Plasmonic Resonator. *Opt. Express* **2010**, *18* (23), 23633–23645.

(2) Yan, W.; Guo, Y.; Beri, D.; Dottermusch, S.; Chen, H.; Richards, B. S. Experimental Determination of Complex Optical Constants of Air-Stable Inorganic CsPbI3 Perovskite Thin Films. *Phys Status Solidi Rapid Res Lett* **2020**, *14* (6), 2000070.

(3) Aftenieva, O.; Schnepf, M.; Mehlhorn, B.; König, T. A. F. Tunable Circular Dichroism by Photoluminescent Moiré Gratings. *Adv. Opt. Mater.* **2021**, *9* (4), 2001280.

(4) Kagan, C. R.; Murray, C. B.; Bawendi, M. G. Long-Range Resonance Transfer of Electronic Excitations in Close-Packed CdSe Quantum-Dot Solids. *Phys. Rev. B* **1996**, *54* (12), 8633–8643.

(5) *Non-Linear Least-Squares Minimization and Curve-Fitting for Python*. https://lmfit.github.io/lmfit-py/ (accessed 2022-06-01).

(6) Makarov, S. V.; Milichko, V.; Ushakova, E. V.; Omelyanovich, M.; Cerdan Pasaran, A.; Haroldson, R.; Balachandran, B.; Wang, H.; Hu, W.; Kivshar, Y. S.; Zakhidov, A. A. Multifold Emission Enhancement in Nanoimprinted Hybrid Perovskite Metasurfaces. *ACS Photonics* **2017**, *4* (4), 728–735.

(7) Hou, S.; Xie, A.; Xie, Z.; Tobing, L. Y. M.; Zhou, J.; Tjahjana, L.; Yu, J.; Hettiarachchi, C.; Zhang, D.; Dang, C.; Teo, E. H. T.; Birowosuto, M. D.; Wang, H. Concurrent Inhibition and Redistribution of Spontaneous Emission from All Inorganic Perovskite Photonic Crystals. *ACS Photonics* **2019**, *6* (6), 1331–1337.

(8) Zhou, X.; Li, M.; Wang, K.; Li, H.; Li, Y.; Li, C.; Yan, Y.; Zhao, Y.; Song, Y. Strong Photonic-Band-Gap Effect on the Spontaneous Emission in 3D Lead Halide Perovskite Photonic Crystals. *ChemPhysChem* **2018**, *19* (16), 2101–2106.

(9) Adamo, G.; Swaha Krishnamoorthy, H. N.; Cortecchia, D.; Chaudhary, B.; Nalla, V.; Zheludev, N. I.; Soci, C. Metamaterial Enhancement of Metal-Halide Perovskite Luminescence. *Nano Lett.* **2020**, *20* (11), 7906–7911.

(10) Wang, H.; Liu, S.-C.; Balachandran, B.; Moon, J.; Haroldson, R.; Li, Z.; Ishteev, A.; Gu, Q.; Zhou, W.; Zakhidov, A.; Hu, W. Nanoimprinted Perovskite Metasurface for Enhanced Photoluminescence. *Opt. Express* **2017**, *25* (24), A1162–A1171.

(11) Zhang, J.; Guo, Q.; Li, X.; Li, C.; Wu, K.; Abrahams, I.; Yan, H.; Knight, M. M.; Humphreys, C. J.; Su, L. Solution-Processed Epitaxial Growth of Arbitrary Surface Nanopatterns on Hybrid Perovskite Monocrystalline Thin Films. *ACS Nano* **2020**, *14* (9), 11029–11039.

(12) Chen, W.; Sha, J.; Yan, K.; Luo, J.; Xu, R.; Yao, D.; Liu, X.; Liao, S.; Zhong, J.; Yang, S.; Yu, Y.; Tong, Y.; Xu, Z.; Lin, Y.-S.; Kao, T.-S. Improvement of Perovskite Photoluminescence Characteristics by Using a Lithography-Free Metasurface. In *International Conference on Optical MEMS and Nanophotonics (OMN)*; **2018**; pp 1–5.

(13) Park, S.; Cho, J.; Jeong, D.; Jo, J.; Nam, M.; Rhee, H.; Han, J. S.; Cho, Y. J.; Ju, B.-K.; Ko, D.-H.; Jang, H. S. Simultaneous Enhancement of Luminescence and Stability of CsPbBr3 Perovskite Nanocrystals via Formation of Perhydropolysilazane-Derived Nanopatterned Film. *Chem. Eng. J.* **2020**, *393*, 124767.

(14) Fang, R.; Lin, Y.-S. Enhancement of Perovskite Photoluminescence Characterizations by Using Lithography-Free Metal Nanostructures. *AIP Advances* **2020**, *10* (6), 065328.

(15) Tian, J.; Adamo, G.; Liu, H.; Klein, M.; Han, S.; Liu, H.; Soci, C. Optical Rashba Effect in a Light-Emitting Perovskite Metasurface. *Adv. Mater.* **2022**, *34* (12), 2109157.

(16) Berestennikov, A. S.; Vakulenko, A.; Kiriushechkina, S.; Li, M.; Li, Y.; Zelenkov, L. E.; Pushkarev, A. P.; Gorlach, M. A.; Rogach, A. L.; Makarov, S. V.; Khanikaev, A. B. Enhanced Photoluminescence of Halide Perovskite Nanocrystals Mediated by a Higher-Order Topological Metasurface. *J. Phys. Chem. C* **2021**, *125* (18), 9884–9890.

(17) Gholipour, B.; Adamo, G.; Cortecchia, D.; Krishnamoorthy, H. N. S.; Birowosuto, Muhammad. D.; Zheludev, N. I.; Soci, C. Organometallic Perovskite Metasurfaces. *Adv. Mater.* **2017**, *29* (9), 1604268.

(18) Tiguntseva, E. Y.; Sadrieva, Z.; Stroganov, B. V.; Kapitonov, Yu. V.; Komissarenko, F.; Haroldson, R.; Balachandran, B.; Hu, W.; Gu, Q.; Zakhidov, A. A.; Bogdanov, A.; Makarov, S. V. Enhanced Temperature-Tunable Narrow-Band Photoluminescence from Resonant Perovskite Nanograting. *Appl. Surf. Sci.* **2019**, *473*, 419–424.

(19) Kessel, A.; Frydendahl, C.; Indukuri, S. R. K. C.; Mazurski, N.; Arora, P.; Levy, U. Soft Lithography for Manufacturing Scalable Perovskite Metasurfaces with Enhanced Emission and Absorption. *Adv. Opt. Mater.* **2020**, *8* (23), 2001627.

(20) Fong, C. F.; Yin, Y.; Chen, Y.; Rosser, D.; Xing, J.; Majumdar, A.; Xiong, Q. Silicon Nitride Nanobeam Enhanced Emission from All-Inorganic Perovskite Nanocrystals. *Opt. Express* **2019**, *27* (13), 18673–18682.

(21) Tiguntseva, E.; Chebykin, A.; Ishteev, A.; Haroldson, R.; Balachandran, B.; Ushakova, E.; Komissarenko, F.; Wang, H.; Milichko, V.; Tsypkin, A.; Zuev, D.; Hu, W.; Makarov, S.; Zakhidov, A. Resonant Silicon Nanoparticles for Enhancement of Light Absorption and Photoluminescence from Hybrid Perovskite Films and Metasurfaces. *Nanoscale* **2017**, *9* (34), 12486–12493.

(22) Baryshnikova, K.; Gets, D.; Liashenko, T.; Pushkarev, A.; Mukhin, I.; Kivshar, Y.; Makarov, S. Broadband Antireflection with Halide Perovskite Metasurfaces. *Laser Photonics Rev.* **2020**, *14* (12), 2000338.

(23) Vancha, A. R.; Govindaraju, S.; Parsa, K. V.; Jasti, M.; González-García, M.; Ballestero, R. P. Use of Polyethyleneimine Polymer in Cell Culture as Attachment Factor and Lipofection Enhancer. *BMC Biotechnol* **2004**, *4*, 23.
